# A Comparative Study of Student Perspectives on Online Versus In-Person Objective Structured Clinical Examination (OSCE) Teaching at a Medical School in London

**DOI:** 10.7759/cureus.86001

**Published:** 2025-06-14

**Authors:** Nimalesh Yogarajah, Aniket Bharadwaj, Amelia Snook, Vashist Motkur

**Affiliations:** 1 Department of Trauma and Orthopaedics, Royal National Orthopaedic Hospital, London, GBR; 2 Department of Trauma and Orthopaedics, Royal Oldham Hospital, Oldham, GBR; 3 School of Medicine, University College London Medical School, London, GBR; 4 Department of Trauma and Orthopaedics, University Hospital Coventry and Warwickshire, Coventry, GBR

**Keywords:** covid-19, medical students, objective structured clinical examination, online teaching, virtual education

## Abstract

Purpose: Objective Structured Clinical Examinations (OSCEs) are widely used in medical education to assess clinical competency through structured stations. During the COVID-19 pandemic, virtual OSCE (vOSCE) teaching became essential due to restrictions on in-person learning. This study compares medical students' perspectives on vOSCE teaching during the pandemic and in-person OSCE (iOSCE) teaching after the pandemic, assessing the role of vOSCE teaching in a postpandemic context.

Methods: Medical students from University College London Medical School participated in vOSCE (2021/2022) or iOSCE (2023), using identical teaching stations. Feedback was gathered through Likert scale questionnaires, and responses were statistically analyzed to identify any significant differences between the cohorts.

Results: One hundred thirty-one iOSCE and 115 vOSCE participants completed feedback. Students showed a clear preference for in-person teaching in areas such as time efficiency (p < 0.001), interaction with tutors and peers (p < 0.001), and future preference for teaching format (p < 0.001). No significant difference was observed in terms of accessibility (p = 0.106). Free-text responses highlighted that in-person sessions were favored for improved interactivity, though some noted that sessions often overran.

Conclusion: iOSCE teaching was favored for better efficiency and interaction, while vOSCE teaching was valued for accessibility. A hybrid approach may be optimal depending on the specific content of the OSCE curriculum.

## Introduction

Medical schools across the UK have been using Objective Structured Clinical Examination (OSCE) for many years, first introduced in the literature by Harden et al. in 1975 [1]. They enable clinical competency and safety assessment in 10-12 stations lasting either five or ten minutes. Core skills are assessed in person, including history taking, patient communication, clinical assessment, practical skills, and prescribing and other medical documentation [1]. In preparation for OSCE examinations, OSCE teaching is often delivered in a low-fidelity, peer-assisted method, combining mock simulation and didactic teaching and feedback [2,3].

During the COVID-19 pandemic and the subsequent introduction of social distancing, most in-person settings were greatly limited, significantly impacting the ability to conduct in-person OSCE (iOSCE) teaching. Most medical teaching transitioned to an online format [4] to mitigate infection risk in all those who participated [5]. Despite the interactive nature of OSCE teaching, it often requires in-person communication between a medical candidate, examiner, and patient role player [1]. It also needed to be converted to a virtual format through online mediums such as “Zoom” [4,6]. There were positives to be gained: studies showed that OSCE teaching and supplementary resources were more easily accessible via online means, reduced travel time, and ran more efficiently [6]. In one study, virtual teaching was also shown to have similar levels of engagement and interactivity to in-person teaching [7]. However, limitations were highlighted in the literature, including the inability to comprehensively practice practical skills and procedures. Moreover, some students were also disrupted due to technological issues such as internet connectivity and audio/visual lag [7]. Following the easing of COVID-19 restrictions and the return to a pre-COVID-19 norm, aspects of medical school teaching shifted back to an in-person format, including OSCE teaching. Though much of medical education has been traditionally delivered in person, there is little literature that investigates whether there is still a role for virtual teaching in the postpandemic world, especially in the OSCE format.

We have previously described the experiences of students undertaking peer-assisted OSCE teaching series in the virtual setting during the pandemic year of 2020-2021 [6]. After COVID-19, we now aim to directly compare the experiences of these students to those of the postpandemic cohort undertaking an in-person iteration of our OSCE teaching series. This OSCE course has been conducted twice via online means in both 2021, published in our previous study [6], and 2022, and then, in person in 2023. This study will seek to compare the data collected from the virtual teaching in the years 2021-2022 to the in-person teaching in the year 2023.

## Materials and methods

This study did not require ethical approval, as per the National Health Service Research Ethics Committee tool. This study did not include a clinical trial and did not collect any personal data. Completion of the feedback survey was optional for participants, and consent was received to use anonymous information to analyze the teaching series.

We describe a descriptive study based on a comparison of feedback from postteaching surveys distributed to students who attended either the online or the in-person teaching series.

The iOSCE teaching series is a continuation of the virtual surgically focused OSCE (vOSCE) teaching designed during the COVID-19 pandemic and delivered in the years 2020-2021 and 2021-2022 at University College London Medical School (UCLMS, via the Surgical Society). The in-person component was carried out from February to May 2023, and the online components described were carried out from February to May 2021 and 2022. Optional post-teaching feedback forms were completed via Google Forms (Google LLC, Mountain View, CA) to assess perceived comparisons of in-person and virtual teaching sessions.

The stations used in this study are the same as those used in our previous study to allow direct comparison (see Appendix 1). They encompassed six common surgical scenarios, focusing on testing clinical knowledge, communication skills, and data interpretation. These topics were tested through history taking, radiological image interpretation, communication skills, prescribing assessments, and the management of an unwell patient.

The execution of the stations within the teaching session was also identical. Students were grouped into threes, rotating through the roles of patient, candidate, and examiner. Stations were either five or ten minutes long. Time was allocated for reading the vignette, the station, and the examiner-candidate feedback at the end.

Per our previous study protocol [6], a "validation" process was conducted. The stations were verified by members of the UCLMS Year-4 Medical Education Faculty and peer-reviewed by fifth-year medical students to ensure that the content of teaching remained in alignment with the medical school curriculum.

In the previously undertaken vOSCE, students received the stations via Zoom (Zoom Video Communications Inc., San Jose, CA) in breakout rooms, which consisted of three students and a tutor to troubleshoot issues. However, in the iOSCE, students attended the teaching session in a university lecture theater and accessed the study materials by scanning QR codes displayed at the front of the room. Only one tutor was required to oversee the whole teaching session.

Participants

As per our previous study [6], fourth-year medical students undertaking the UCLMS medical degree, in their first year of clinical placement attachments, were offered this teaching series and invited to complete postteaching feedback. There were no exclusion criteria.

The feedback surveys recorded participants’ perceptions on efficiency/accessibility, clarity of instructions, quality of tutor-peer interaction, and preferences for the method of teaching delivery.

Data source/measurement

Feedback questionnaires were asked to be completed by students after the penultimate station on a Google (Google LLC, Mountain View, CA) form, which was sent on Zoom in our previous study and accessed via a QR code in this study. It consisted of a 5-point Likert scale asking to ‘’strongly agree,’’ ‘’agree,’’ ‘’neutral,’’ ‘’disagree,’’ or ‘’strongly disagree’’ with various statements and a free-text field for further comments. As the two cohorts’ (online and in-person teaching participants) feedback was being recorded, two feedback forms were used: one for online and one for in-person teaching cohorts (Appendix 2). However, as students were asked to compare their experiences from their format of teaching to previous experiences of the other (online vs. in-person teaching in the previous study and vice versa for this study), questions were reworded appropriately to account for this difference.

Feedback forms were optional for students though completing the form required filling out all relevant sections; therefore, all forms were fully completed, ensuring no data gaps during analysis.

Statistical analysis

Our study will compare the feedback collected from the in-person teaching in 2022-2023 and the compiled dataset from the two online teaching sessions from 2020 to 2022 (raw data for all three of these cohorts can be found in Appendix 3). In particular, the "extent of agreement" with the questions asked in the Likert scale feedback form was compared across the cohorts (i.e., how much the respondents selected "agree" or "strongly agree" to the given statements). This will be used as an indicator of participants' preferences for different OSCE formats.

Given the ordinal nature of these respondent data, a chi-square analysis was used to assess the statistical significance of any differences noted across the cohorts (iOSCE and vOSCE). Differences were considered significant if they met a p value of <0.05. Statistical analyses were conducted using Python (version 3.12.4, Python Software Foundation, Wilmington, DE) and the SciPy library (version 1.14.1). Chi-square tests of independence were performed, and a p value of <0.05 was considered statistically significant.

## Results

The total number of respondents was 246: 131 respondents from the iOSCE cohort responded to the questionnaire compared to the 115 vOSCE students (Table 1).

**Table 1:** Quantitative Likert Scale responses from feedback questionnaires given to University College London medical students. Data show comparison from the vOSCE sessions of 2021/2022 to the iOSCE sessions of 2023 vOSCE: virtual Objective Structured Clinical Examination; iOSCE: in-person Objective Structured Clinical Examination

Item	Percentage of iOSCE respondents answering “agree” or “strongly agree” (n = 131)	Percentage of vOSCE respondents answering “agree” or “strongly agree” (n = 115)
I found this format of teaching to be more time-efficient	92.4%	65.2%
I found this format of teaching more accessible	84.7%	75.7%
I found it easier to interact with tutors in this format	93.1%	40.9%
I found it easier to communicate with my peers in person in this format	93.1%	33.9%
I would prefer this format of teaching in the future	96.2%	42.6%

Our data revealed a significant preference among students in the iOSCE cohort for the format's efficiency, ease of interaction with tutors, communication with peers, and future preference.

Efficiency

There was a significant difference observed for the statement "I found this format of teaching more time-efficient," with a higher agreement in the in-person cohort, χ²(1) = 26.33, p < 0.001. There was also a difference in the efficiency of teaching, with students in the iOSCE cohort significantly more likely to find it easier to interact with tutors, χ²(1) = 75.19, p < 0.001.

Communication

Concerning communication with peers, there was a significant preference for in-person communication with peers over the virtual format, χ²(1) = 92.28, p < 0.001. With regard to students preferring the said format for future teaching, a significant preference was expressed for the in-person format for future teachings, χ²(1) = 83.11, p < 0.001.

Accessibility

No significant difference was observed for the statement "I found this format of teaching more accessible," suggesting that students did not discern a noticeable difference in accessibility between the two formats, χ²(1) = 2.61, p = 0.106.

Overall preference

Overall, there was a significant difference reflecting a preference for iOSCE teaching over vOSCE teaching, χ²(1) = 83.02, p = 0.001.

Positive free-text responses (Table 2) also supported the data, suggesting a preference in general for in-person teaching for OSCE revision. It also suggested that in-person teaching was more interactive and easier to engage with and participate in. There was also the added benefit of being more able to choose who to practice with in simulation scenarios.

**Table 2:** Key contents of positive free-text responses OSCE: Objective Structured Clinical Examinations

Topic of comment	Example of a positive comment
General preference	In-person is far better for teaching something as practical as OSCEs
	It was great to have face-to-face teaching
Interactivity	Easier to ask questions
	It is more interactive than online
Delivery of teaching	Sometimes it is more intimidating online to ask questions, especially if a session is being recorded, so I enjoyed how much more interactive in-person teaching is
	I could choose to practice with my friends
	In-person teaching feels more engaging

Negative free-text responses (Table 3) were focused on teaching delivery. In particular, the in-person teaching sessions sometimes overran, and venue choice proved to be an important factor in how effectively a teaching session was received.

**Table 3:** Key contents of negative free-text responses

Topic of comment	Example of a negative comment
Delivery of teaching	The room layout is not conducive to learning; perhaps an open room would be better than a lecture theater
	A different venue to allow easier practice
	Teaching overran
	Felt rushed toward the end as we were overrunning

## Discussion

Overall, students significantly favor the iOSCE format of the surgical OSCE in terms of efficiency, interaction with tutors, and peer communication. The notable exception is accessibility, where no significant preference was detected. This indicates a possible area where virtual formats may be comparable to in-person experiences. A variety of factors could explain these differences.

Running educational sessions online requires many more steps to begin the engagement process, e.g., finding the correct link, logging on to the application, ensuring a strong internet connection, confirming microphones and cameras are functioning correctly, and moving between the breakout rooms and the main "room." Although these steps are minor, they may be perceived to be less efficient than an in-person session, where, once the student is physically in the room, the communication can start, as opposed to waiting for all the steps to be completed before starting. An iOSCE environment allows the educational organizers to standardize the environment for the students, such as by booking an appropriate private study room that is free from distractions, which is not possible in a virtual setting. Locations that students use to engage in vOSCE sessions cannot be standardized for levels of distraction, privacy, or audio-visual backgrounds that may negatively affect other participants. This incongruence in virtual teaching settings for all participants could be the reason why in-person teaching sessions are seen to be more efficient. An environment with distractions could also impede the students' motivation to learn. Indeed, studies such as Kaur et al.’s assessment [8] have noted that online learning can be associated with a lack of motivation. This can result in reduced engagement and, ultimately, cause the teaching session to run less efficiently and streamlined. Students' motivation is important for the smooth running of these sessions, especially in an online setting.

The vOSCE cohort may have found it harder to interact with both tutors and peers due to the audio-visual lag that is present on online channels. A disparity in the quality of internet connections across participants may have prevented seamless communication between those engaging in the session. By contrast, iOSCE students will not have faced this issue, where there is no lag in real-time communication, making the transfer of information more seamless. As highlighted in the Motkur et al. study [6], in-person interaction has a more valuable human component and allows the exchange of many more subtle communication cues, such as nuanced body language and microexpressions, aiding the students in developing their interpersonal skills. Although technology is progressing well with video and voice interfaces, it still cannot provide a realistic interface that competes with the in-person experience. A paper looking at in-person and remote learning also shed light on similar points, such as online participants experiencing "Zoom fatigue" and being interested in an in-person interaction with their colleagues and teachers [9]. However, with virtual and mixed reality getting increased exposure and investment, the transfer of such technology into the medical educational field may make the comparison compelling.

No significant difference was noted in perceived accessibility between the vOSCE and iOSCE cohorts. This is surprising as the online platform is widely seen to be more accessible due to convenience in time and location [10]. Certain aspects of OSCE teaching are not accessible online. For example, one of the OSCE stations involved the clinical assessment of an unwell patient. Although a student can verbally state how they would manage the patient in that scenario, they cannot physically carry out any actions through a virtual format, therefore limiting the ability to be appropriately assessed in that clinical scenario. This was also echoed in a paper by Gheshlagh et al. [11], where online learning was inadequate in providing adequate exposure and development of practical skills.

Online teaching also requires proficiency in technology (such as being able to use a platform such as Zoom, or navigating online material on Google Drive), access to software/hardware, and online connectivity to facilitate this. If there is a lack of any of these, then accessing teaching online becomes more difficult and less accessible. However, at the same time, in-person teaching relies on appropriate venue choices: a lecture theater may be beneficial for didactic teaching but not for interactive group-based OSCE simulation.

It is also important to be aware of potential limitations. This is a single-center study where the results may show biases toward the teaching methods of the particular medical school from where data were collected. Extending data collection to include multiple medical schools (and, therefore, teaching styles and curricula) may provide a more complete image of medical student perceptions of in-person and vOSCE teaching. There may also be merit in testing the same cohort of students in both the iOSCE and vOSCE environments to provide more validity in the comparison of the teaching methods. However, we believe that, given that both cohorts of students (albeit different people) were fourth-year students at the time, encountering the curriculum for the first time, they still provided comparable conditions in each cohort. There may also be a bias toward the positive feedback received for online teaching during the COVID-19 period, due to it being mandatory given social distancing rules at the time. Students who attended the iOSCE teaching in the postpandemic setting may have had views biased against virtual teaching, given that it was more optional at the time of their studies.

## Conclusions

Modern technological advancements have meant that aspects of society, work, and education are increasingly being held in a virtual or online platform. This has only been accelerated by the recent COVID-19 pandemic. However, there is still clear evidence that, with education and learning, the in-person setting has clear advantages. This study showed that despite the increase in exposure to online education, students prefer iOSCE education for the values it brings with in-person interaction and teaching efficiency. Crucially, they still voiced a preference for this format in future sessions.

There may still be a role for online teaching, especially with its main advantage of accessibility at any time and from anywhere. Determining which setting best suits a teaching series should depend on the content that is being taught and how it would best be learned and assessed. Within medical education, there is a variety in the type of content taught, from didactic teaching to simulation teaching, for each of which online and in-person teaching may have their relative advantages.

Future studies may benefit from recruiting multicenter cohorts and using objective comparisons, such as OSCE performance scores or fluency of practical skills following each teaching platform.

## Figures and Tables

**Figure 1:**
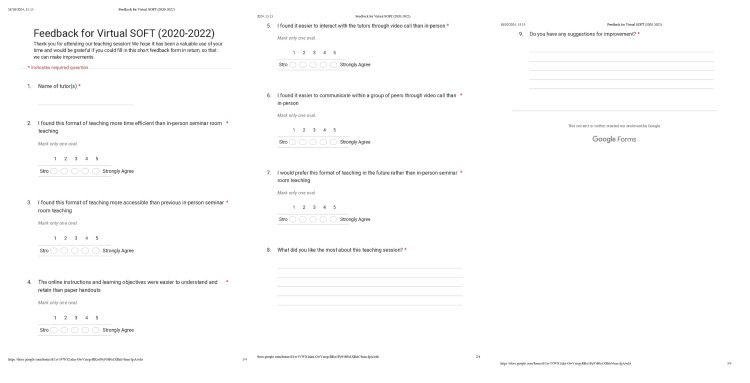
Feedback form for participants for the Virtual OSCE (vOSCE) from 2020-2022

**Figure 2:**
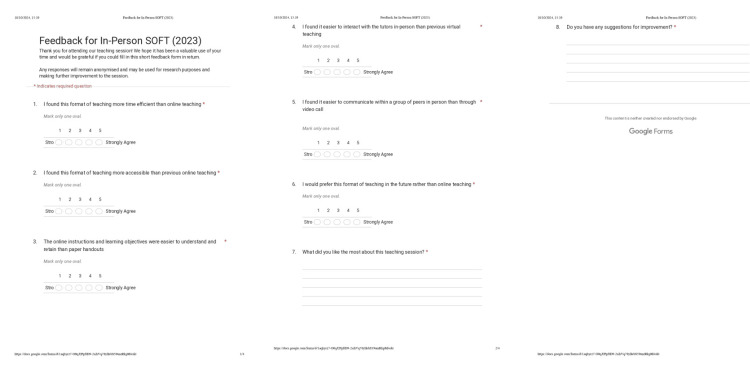
Feedback form for participants for the In-Person OSCE (iOSCE) from 2023

## Appendices

Appendix 1

**Table 4:** Description of the content of the objective structured clinical examination stations

Station no.	Clinical context	Task 1	Task 2
1	Dysphagia	Focused history-taking (five minutes)	Communication with medical professionals using the “situation, background, assessment, recommendation” framework (three minutes)
2	Fractured neck of femur	Interpreting and explaining a radiograph to a nursing student, including initial and definitive management (five minutes)	Safe prescription of regular medications and analgesia using the World Health Organization Analgesic Ladder (five minutes)
3	Scaphoid fracture	Interpreting and explaining a radiograph to a patient, including answering questions on further management (five minutes)	-
4	Stomas	Matching clinical vignettes of conditions requiring ileostomy or colostomy and describing the differentiating features of each (three minutes)	Analyzing a fluid balance chart and safe prescription of replacement and maintenance fluids (five minutes)
5	Septic arthritis	Focused history-taking and conveying an initial management plan (five minutes)	-
6	Postoperative sepsis	Assessment of an acutely ill patient using the “airway, breathing, circulation, disability, exposure” approach (10 minutes)	-

Appendix 2

Appendix 3

**Table 5:** Raw data sets for questionnaire feedback collected from vOSCE participants in 2020-2022 and iOSCE participants in 2022-2023 vOSCE: virtual Objective Structured Clinical Examination; iOSCE: in-person Objective Structured Clinical Examination

Likert scale datasets for 2020-2023	Strongly agree	Agree	Neutral	Disagree	Strongly disagree
vOSCE: 2020/2021 (n = 62)					
Time efficiency: I found this format of teaching more time efficient than in-person seminar room teaching	19	21	10	11	1
Accessibility: I found this format of teaching more accessible than the previous in-person seminar room teaching	24	18	10	6	4
Interaction: I found it easier to interact with the tutors through video call than in-person	15	12	17	10	8
Communication: I found it easier to communicate with a group of peers through a video call than in-person	11	10	19	13	9
Preference: I would prefer this format of teaching in the future rather than in-person seminar room teaching	12	13	17	15	5
vOSCE 2021/2022 (n = 53)					
Time efficiency: I found this format of teaching more time efficient than in-person seminar room teaching	21	14	11	7	0
Accessibility: I found this format of teaching more accessible than the previous in-person seminar room teaching	28	17	5	3	0
Interaction: I found it easier to interact with the tutors through video call than in-person	7	13	13	14	6
Communication: I found it easier to communicate with a group of peers through a video call than in-person	5	13	11	6	8
Preference: I would prefer this format of teaching in the future rather than in-person seminar room teaching	13	11	10	14	5
iOSCE 2022/2023 (n = 131)					
Time efficiency: I found this format of teaching more time-efficient than online teaching	97	24	9	1	0
Accessibility: I found this format of teaching more accessible than previous online teaching	81	30	17	3	0
Interaction: I found it easier to interact with the tutors in-person than in previous virtual teaching	96	26	9	0	0
Communication: I found it easier to communicate within a group of peers in person than through a video call	109	13	7	2	0
Preference: I prefer this format of teaching in the future rather than online teaching	106	20	5	0	0
